# Fibroblast Growth Factor-23-Klotho Axis in Cardiorenal Syndrome: Mediators and Potential Therapeutic Targets

**DOI:** 10.3389/fphys.2021.775029

**Published:** 2021-11-15

**Authors:** José Alberto Navarro-García, Laura González-Lafuente, María Fernández-Velasco, Luis M. Ruilope, Gema Ruiz-Hurtado

**Affiliations:** ^1^Cardiorenal Translational Laboratory, Institute of Research i + 12, Hospital Universitario 12 de Octubre, Madrid, Spain; ^2^IdiPAZ Institute for Health Research/CIBER-CV, Hospital Universitario La Paz, Madrid, Spain; ^3^CIBER-CV, Hospital Universitario 12 de Octubre, Madrid, Spain; ^4^School of Doctoral Studies and Research, European University of Madrid, Madrid, Spain

**Keywords:** FGF-23, Klotho, cardiorenal syndrome, chronic kidney disease, acute kidney injury, dialysis, heart failure

## Abstract

Cardiorenal syndrome (CRS) is a complex disorder that refers to the category of acute or chronic kidney diseases that induce cardiovascular disease, and inversely, acute or chronic heart diseases that provoke kidney dysfunction. There is a close relationship between renal and cardiovascular disease, possibly due to the presence of common risk factors for both diseases. Thus, it is well known that renal diseases are associated with increased risk of developing cardiovascular disease, suffering cardiac events and even mortality, which is aggravated in those patients with end-stage renal disease or who are undergoing dialysis. Recent works have proposed mineral bone disorders (MBD) as the possible link between kidney dysfunction and the development of cardiovascular outcomes. Traditionally, increased serum phosphate levels have been proposed as one of the main factors responsible for cardiovascular damage in kidney patients. However, recent studies have focused on other MBD components such as the elevation of fibroblast growth factor (FGF)-23, a phosphaturic bone-derived hormone, and the decreased expression of the anti-aging factor Klotho in renal patients. It has been shown that increased FGF-23 levels induce cardiac hypertrophy and dysfunction and are associated with increased cardiovascular mortality in renal patients. Decreased Klotho expression occurs as renal function declines. Despite its expression being absent in myocardial tissue, several studies have demonstrated that this antiaging factor plays a cardioprotective role, especially under elevated FGF-23 levels. The present review aims to collect the recent knowledge about the FGF-23-Klotho axis in the connection between kidney and heart, focusing on their specific role as new therapeutic targets in CRS.

## Introduction

Cardiorenal syndrome (CRS) is defined as a complex pathological disorder that involves the kidneys and heart, whereby acute or chronic dysfunction in one organ may induce acute or chronic dysfunction in the other (Ronco et al., 2010). The presence of both cardiovascular and renal disease increases the risk of suffering cardiovascular events and death, even after adjustment for traditional cardiovascular risk factors (Gansevoort et al., 2013). Depending on the origin of the pathology, CRS can be classified into five different subtypes: acute CRS, whether the acute worsening of the heart function leads to a renal dysfunction or kidney injury (type 1 CRS or CRS1); chronic CRS, where the chronic heart dysfunction induces renal dysfunction or kidney injury (type 2 CRS or CRS2); acute reno-cardiac syndrome, which is an acute kidney injury (AKI) that leads to a heart dysfunction (type 3 CRS or CRS3); chronic reno-cardiac syndrome, when the existence of chronic kidney disease (CKD) leads to heart dysfunction (type 4 CRS or CRS4); and secondary CRS, where another pathology induces the development of heart and kidney disease simultaneously (type 5 CRS or CRS5) (Ronco et al., 2010).

## Cardiovascular Events and Mortality in Renal Disease

It is well established that renal disease is associated with a high risk of cardiovascular disease (Gansevoort et al., 2013; Legrand and Rossignol, 2020). Compared to the healthy population, renal patients present greater all-cause and cardiovascular mortality (van der Velde et al., 2011; Odutayo et al., 2017), especially end-stage renal disease (ESRD) patients (de Jager et al., 2009). Thus, more than 50% of renal patients die due to cardiovascular complications even before reaching ESRD. Premature deaths are also very common before and during dialysis cycles (Go et al., 2004), with a 10–20-fold greater cardiovascular mortality risk in dialysis patients than in the general population (de Jager et al., 2009). This increased cardiovascular mortality might be linked to the fact that more than half of ESRD patients develop cardiac arrhythmias (Hsieh et al., 2020; Kim et al., 2021) or heart failure (HF) (House et al., 2019). Renal patients usually develop left ventricular hypertrophy (LVH) (Middleton et al., 2001), atrial fibrillation (AF) (Hsieh et al., 2020), HF (Schefold et al., 2016; Odutayo et al., 2017), ischemic heart disease, and ventricular tachycardia (Kim et al., 2021) which are related to increased risk of mortality in these patients. In this context, there are some kidney disease-related pro-arrhythmogenic risk factors that might predispose these patients to suffer arrhythmias and cardiac structure alterations such as variation in fluxes of key electrolytes, metabolic acidosis, and the presence of uremic toxins (Tang et al., 2015; Di Lullo et al., 2019).

On the other hand, the two main factors that define renal disease—albuminuria and decreased estimated glomerular filtration rate (eGFR)—are both independent risk factors for the prediction of cardiovascular events and death (van der Velde et al., 2011; Gansevoort et al., 2013). Thus, lower eGFR and higher albumin-to-creatinine ratio (ACR) have been associated with a significant increment to cardiovascular mortality (Ruilope and Bakris, 2011; van der Velde et al., 2011; Gansevoort et al., 2013). Both eGFR and ACR values are even more useful than other traditional risk factors as predictors of incident cardiovascular events such as HF (Matsushita et al., 2015). In addition, as renal function declines, new risk factors appear that contribute to renal dysfunction while also increasing the risk of cardiovascular damage (Ruilope and Bakris, 2011). Among these are biological aging, hypertension, diabetes mellitus, atherosclerosis, endothelial dysfunction, accumulation of uremic toxins, and mineral bone disorders (MBDs) (Ruiz-Hurtado and Ruilope, 2014; Ruiz-Hurtado et al., 2016). The development of cardiovascular disease associated with the uremic milieu is named uremic cardiomyopathy. Uremic cardiomyopathy was first described in Bailey et al. (1967) who observed cardiac hypertrophy and its relation with diet in CKD patients with highly increased creatinine and urea serum levels. Despite the clear clinical association between kidney failure and cardiac damage and *vice versa*, the underlying mechanisms connecting both systems are not well understood.

### Left Ventricular Hypertrophy

LVH is one of the most common cardiac alterations described in renal patients (Middleton et al., 2001; Faul et al., 2011). LVH can be associated with ischemic heart disease, diastolic dysfunction, and a decompensated situation that can induce HF over the long-term (Harnett et al., 1995), as it increases the probability of cardiac arrhythmias (Rantanen et al., 2020) that are associated with a decreased survival rate in renal patients (Banerjee, 2016). The development of LVH in renal patients might be the consequence of maintained pressure overload and hypertension, both of which are frequently observed in these patients. Shen et al. (2020) recently proposed cystatin C secretion by cardiomyocytes in response to pressure overload as a possible promoter of cardiac hypertrophy in renal patients. Other studies have focused on the alteration of mineral bone components in renal patients as an important factor in the development of LVH, describing a direct pro-hypertrophic effect of fibroblast growth factor (FGF)-23 on the heart (Faul et al., 2011). However, other authors have proposed the FGF-23-mediated activation of the cardiac renin-angiotensin-aldosterone system (RAAS) as the inductor of cardiac hypertrophy and fibrosis in renal patients (Böckmann et al., 2019). More studies are needed to elucidate which of these factors is the early inducer of cardiac hypertrophy in renal patients; that will facilitate the determination of whether there is a main causative factor, or whether it is the combination of all factors that is responsible for the deleterious cardiac remodeling.

### Heart Failure

HF is the main cardiovascular complication and the leading cause of mortality in renal patients (Tuegel and Bansal, 2017; Go et al., 2018), especially in ESRD patients (Bansal et al., 2017). HF consists of the heart inability to supply the peripheral tissue demands with enough blood and oxygen. HF patients are divided according to the ejection fraction (EF) as patients with systolic HF, who present reduced EF (HF-rEF), and patients with diastolic HF, with preserved systolic function but compromised diastolic function (preserved EF, HF-pEF). HF-rEF is a consequence of an impairment of left ventricular contractility, usually linked to eccentric remodeling with chamber dilatation. By contrast, HF-pEF is characterized by an impaired ventricular relaxation and filling as consequence of the concentric remodeling. In most cases, HF appears due to a subjacent myocardial disease as consequence of a myocardial ischemia. However, HF can also be developed from a concomitant disease such as renal disease. In this line, the risk of HF development significantly increases in AKI patients over the long term (Gammelager et al., 2014; Go et al., 2018). It has been described that stage 3 CKD patients present a threefold higher risk of HF development compared to healthy population (Kottgen et al., 2007). CKD patients can develop either diastolic or systolic HF. It has been found that the majority of ESRD patients display HF-pEF with diastolic dysfunction and presence of LVH, while HF-rEF is visible only in the minority of these patients (Antlanger et al., 2017). Notably, it is the HF-pEF phenotype that represents a higher mortality risk in renal patients (Ahmed et al., 2007). It is further important to remark that HF development begins even in the early stages of CKD. Indeed, assessment of a large group of HF patients has shown that diastolic dysfunction, systolic dysfunction, and mortality increased in parallel with the progression of CKD (Unger et al., 2016). This might be explained by the fact that HF is a status of low cardiac output that diminishes the effective glomerular filtration pressure, decreasing eGFR and promoting the development or progression of CKD. At the experimental level, a significant reduction in EF was observed in CKD mice following 5/6 nephrectomy (Navarro-García et al., 2020). In an experimental AKI model, mice developed diastolic dysfunction with reduced EF (Hu et al., 2017). However, other authors have shown more recently that AKI mice develop HF with preserved EF (Fox et al., 2019; Soranno et al., 2021). In spite of the differences observed, which are possibly based on the method used to induce renal disease and the time of renal damage progression, renal disease mouse models have clearly shown HF development. It is likely that kidney dysfunction not only plays an important causative role in the development and progression of HF, but also acts as a marker of HF severity.

### Arrhythmias

In dialysis patients, arrhythmia is a common symptom frequently observed in the inter-dialytic interval (Roy-Chaudhury et al., 2018). Bradycardias and asystole are the more common arrhythmias described in the inter-dialytic interval, while ventricular tachycardia and AF have been found during the dialysis procedure (Roy-Chaudhury et al., 2018; Hsieh et al., 2020). However, it is not easy to determine the main factor triggering the arrhythmia responsible for sudden cardiac death (SCD) in dialysis patients; this is because SCD usually happens outside of the dialysis unit during the inter-dialytic interval. As ventricular arrhythmia is possibly the main cause of SCD in the general population (Priori et al., 2001), this fatal event might be linked to the high prevalence of cardiac deaths found in CKD and ESRD patients (Genovesi et al., 2013; Coll et al., 2018). Recent studies have pointed to FGF-23 as an important inducer factor of ventricular arrhythmias (Navarro-García et al., 2019).

## Mineral Bone Disorders and Cardiorenal Syndrome

Mineral metabolism disturbances are closely connected with both AKI (Leaf and Christov, 2019) and CKD (Rouached et al., 2008). MBDs contribute to the augmented morbidity and mortality observed in renal patients (Block et al., 2004). MBD is aggravated with the decrease of renal function. MBDs include changes in serum calcium (Ca^2+^) concentration, increased serum phosphate levels (hyperphosphatemia), reduced serum levels of active vitamin D, secondary increase of parathyroid hormone (PTH), augmented FGF-23 systemic levels, and reduced levels of the antiaging factor Klotho (Wang et al., 2018). Mineral bone components are regulated by important negative and positive feedback loops between them (Navarro-García et al., 2018). Thus, the systemic levels of any one of these components depend on the levels of the others, with the objective of maintaining an adequate phosphate and Ca^2+^ homeostasis. However, the loss of renal function disrupts these feedback loops. Moreover, changes in any one of these parameters misbalance the feedback loops, altering the circulating levels of the others and inducing significant effects on remote organs such as the heart. In the context of renal disease, the increased levels of phosphate, increased PTH, vitamin D deficiency, Klotho deficiency, and augmented FGF-23 levels might facilitate cardiovascular events, including structural alterations like LVH (Faul et al., 2011), cardiac dysfunction such as HF (Wannamethee et al., 2014), and rhythm alterations such as AF (Mathew et al., 2014) or ventricular arrhythmia (Navarro-García et al., 2019, 2020).

### Vitamin D and Parathyroid Hormone

Ca^2+^ homeostasis is chiefly regulated by 1,25-dihydroxycholecalciferol D3 (or vitamin D) and PTH. A reduction of serum Ca^2+^ concentration activates the synthesis of PTH in the parathyroid gland. PTH binds to PTH receptors (PTHR) in the kidney, stimulating Ca^2+^ reabsorption but also increasing vitamin D synthesis. Vitamin D binds to vitamin D receptors (VDR) on the surface of enterocytes in the intestinal tract, increasing dietary Ca^2+^ absorption by augmenting the expression of the transient receptor potential cation channel subfamily V member 6 and plasma membrane Ca^2+^-ATPase (Christakos et al., 2010). Furthermore, the binding of vitamin D to VDR of the parathyroid gland inhibits PTH synthesis, which constitutes an important feedback mechanism related to Ca^2+^ homeostasis (Navarro-García et al., 2018). The loss of renal function is frequently related to vitamin D deficiency (Wolf, 2010) and increased synthesis of PTH (secondary hyperparathyroidism) (Madsen et al., 1981; Naveh-Many and Volovelsky, 2020).

Reduced serum vitamin D levels in renal patients arise due to impaired activity of the renal enzyme 1-α-hydroxylase, which transforms 25-hydroxyvitamin D3 into the active hormone form of vitamin D, 1,25-dihydroxycholecalciferol D3. However, vitamin D and PTH are not only important for Ca^2+^ homeostasis, but also play an important role in phosphate homeostasis. Serum PTH acts on type II sodium/phosphate cotransporters to reduce renal phosphate reabsorption (Kronenberg, 2002). At the gut level, vitamin D binding to VDR on enterocytes also increases the expression of the sodium-dependent phosphate transporter Pit-2, facilitating phosphate absorption (Katai et al., 1999). It is well known that changes in both vitamin D and PTH are related to important cardiovascular alterations, especially in renal patients. Thus, vitamin D deficiency has been linked to a higher risk of cardiac hypertrophy, LV dysfunction, HF development and mortality (Bodyak et al., 2007; Anderson et al., 2010; Gotsman et al., 2012). In this line, several studies have demonstrated that treatment with vitamin D analogs such as paricalcitol can reduce vascular calcification (Anis et al., 2020) and LVH (Bodyak et al., 2007; Leifheit-Nestler et al., 2017) under uremic conditions. Moreover, paricalcitol treatment has also been shown to impede the progression of HF by improving adverse Ca^2+^ mishandling even in the absence of renal disease (Tamayo et al., 2020). In addition, PTH disorders have been related to hypertension, valve calcification, HF (Khouzam et al., 2006), arrhythmias (Curione et al., 2010), and mortality in renal patients (Molina et al., 2021).

### Phosphates

Systemic phosphate levels appear to increase in renal patients due to the loss of renal function (Jung et al., 2018; Bacchetta et al., 2020). High phosphate levels have been related to mortality in renal patients (Merhi et al., 2017; Moon et al., 2019), mainly in those undergoing dialysis (Owaki et al., 2018), but also in the general population (Chang and Grams, 2014). Phosphate homeostasis depends on the counterbalance between dietary phosphate intake, phosphate mobilization from bone, and renal phosphate excretion. Thus, renal dysfunction carries an important increment of serum phosphate levels. Classically, serum phosphates levels have been considered the main cause of increased risk of cardiovascular disease among renal patients, including the higher risk of mortality (Palmer et al., 2011; Scialla and Wolf, 2014; Moon et al., 2019). Phosphates have been described as promoting vascular calcification in renal patients (Cozzolino et al., 2019). Furthermore, phosphate levels have been linked to left ventricular remodeling in renal patients (Zou et al., 2016).

### Fibroblast Growth Factor-23

The main physiological regulator of serum phosphate levels is FGF-23, which acts as a phosphaturic hormone (Seiler et al., 2009). Phosphates have traditionally been established as the main target to control in renal disease, with FGF-23 having been considered a “*secondary player*” that has the apparently unique role of reducing serum phosphate levels. However, FGF-23 has recently emerged as a new and direct factor in the context of cardiac damage. It has accordingly been described that increased FGF-23 levels are the first alteration observed in renal patients, even earlier than elevated phosphate serum levels (Wolf, 2012; Christov et al., 2013). FGF-23 is a hormone mainly synthesized by osteocytes and osteoblasts in long bones to control phosphate homeostasis and serum levels of vitamin D and PTH (Navarro-García et al., 2018). The principal stimulus for FGF-23 synthesis is an increase in systemic phosphate levels due to high phosphate diet uptake (Antoniucci et al., 2006) or decreased renal phosphate excretion, as occurs in renal patients (Larsson et al., 2003).

**FIGURE 1:**
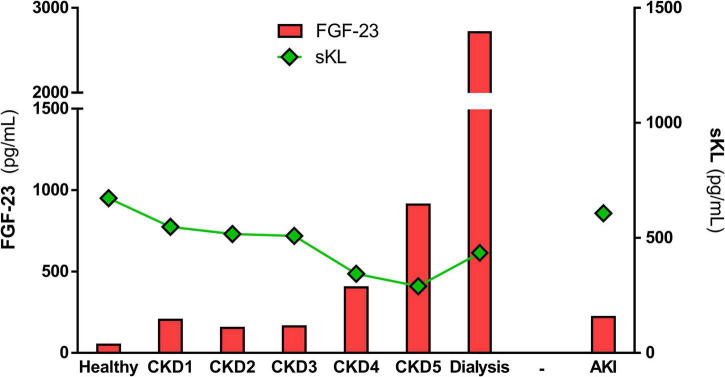
Mean values of FGF-23 and sKL in healthy population and alongside renal disease. The graph represents the mean values of serum FGF-23 and sKL levels described in the different renal cohorts included in Tables 1, 2.

FGF-23 effect is mediated by binding to one of the FGF receptor isoforms (FGFR1-4). FGF-23 binding to FGFR requires the presence of a co-factor due to FGF-23’s low affinity for all FGFRs (Yu et al., 2005). The FGFR1 co-factor in the kidney is the transmembrane protein Klotho (Kurosu and Kuro-o, 2009), which reveals the importance of Klotho expression for FGF-23 phosphaturic effects. All organs with FGFR and Klotho expression are susceptible to FGF-23 effects. However, Urakawa et al. (2006) proposed that the only FGFR isoform capable of combining with Klotho to induce FGF-23 signaling is FGFR1, being able FGF-23 to bind to the other FGFRs in a Klotho-independent manner. In this sense, FGF-23 can also act independently of Klotho through its binding to FGFR4, as occurs in Klotho-free organs such as the heart (Faul et al., 2011; Grabner et al., 2015). In renal patients, renal excretion of phosphates decreases due to the reduction of renal Klotho expression, increasing serum phosphate levels and stimulating a maintained pathological synthesis of FGF-23 (Gutiérrez et al., 2008). In fact, renal patients present extremely high serum FGF-23 levels compared to healthy people. These levels increase as renal function declines (Isakova et al., 2020), reaching the highest levels in patients undergoing dialysis (see Figure 1 and Table 1). Serum FGF-23 levels increase from ∼40 pg/mL in healthy subjects to ∼200 pg/mL in AKI patients and stage 1 CKD patients. Throughout CKD progression, serum FGF-23 continues to increase due to the progressive loss of renal function, reaching levels 20-fold higher than those described in the healthy population. It is important to note that FGF-23 remains at similar values in the early stages of CKD (CKD1-3), while the increment of FGF-23 is greater from stage 4 CKD to ESRD. The highest serum FGF-23 levels are found in those renal patients undergoing dialysis treatment, with values above 2,000 pg/mL. The phosphaturic effect of FGF-23 tries to protect against the deleterious actions of increased serum phosphates aggravated by renal dysfunction. However, it is impossible to efficiently remove phosphates from blood in advanced-stage CKD and in dialysis patients. As a result, serum FGF-23 levels are necessarily up to 1,000 times higher in these patients than among healthy people (Wolf, 2012), and these enormous FGF-23 levels can have deleterious pathological consequences on off-target organs.

**TABLE 1:** Mean values of serum FGF-23 levels measured in pg/mL in healthy subjects and different renal patients.

Population	FGF-23 values (pg/mL)	References
Healthy	42.7 ± 18.6	Yamazaki et al., 2010; Chathoth et al., 2015; Leaf et al., 2018; Neyra et al., 2019; Rodríguez-Ortiz et al., 2020; Shardell et al., 2020; Matei et al., 2021; Salam et al., 2021
AKI	220.9 ± 126.8	Leaf et al., 2018; Rygasiewicz et al., 2018; Fayed et al., 2019; Neyra et al., 2019
CKD1	203.5 ± 346.0	Evenepoel et al., 2010; Kim et al., 2013; Iio et al., 2019; Khodeir et al., 2019; Bielesz et al., 2020
CKD2	153.3 ± 296.0	Evenepoel et al., 2010; Kanbay et al., 2010; Kim et al., 2013; Iio et al., 2019; Khodeir et al., 2019; Bielesz et al., 2020; Shardell et al., 2020; Kritmetapak et al., 2021
CKD3	162.7 ± 264.8	Evenepoel et al., 2010; Kim et al., 2013; Chathoth et al., 2015; Bouma-de Krijger et al., 2019; Khodeir et al., 2019; Iio et al., 2019; Ramalho et al., 2019; Bielesz et al., 2020; D’Arrigo et al., 2020; Hughes-Austin et al., 2020; Jovanovich et al., 2021; Kritmetapak et al., 2021
CKD4	402.3 ± 537.1	Kim et al., 2013; Chathoth et al., 2015; Khodeir et al., 2019; Iio et al., 2019; Bielesz et al., 2020; Chen et al., 2021; Kritmetapak et al., 2021
CKD5	911.0 ± 640.4	Kim et al., 2013; Chathoth et al., 2015; Khodeir et al., 2019; Iio et al., 2019; Bielesz et al., 2020; Chen et al., 2021; Kritmetapak et al., 2021; Salam et al., 2021
Dialysis	2713.0 ± 1294.0	Fukasawa et al., 2014; Lima et al., 2014; Otani-Takei et al., 2015; Damasiewicz et al., 2018; Kulicki et al., 2019; Chan et al., 2020; Takashi et al., 2020

*Mean values ± SD are obtained as the mean of the values found in the different cohorts studied. Clinical studies in which FGF-23 levels were measured as pg/mL were included in this table. These studies were carried out on diabetic or cardiovascular patient cohorts, with pediatric cohorts excluded.*

Recent studies have evidenced adverse effects of FGF-23 on the heart. In this sense, elevated serum FGF-23 levels have been associated with cardiovascular events in early CKD patients (Isakova et al., 2011; Ix et al., 2012) and are also related to increased all-cause and cardiovascular mortality (Ärnlöv et al., 2013), especially in ESRD patients (Scialla et al., 2014) but also in the general population (De Jong et al., 2021). It has been observed that renal patients who experience faster elevations of FGF-23 serum levels tend to have a higher risk of death (Isakova et al., 2018). Increased serum FGF-23 levels have also been associated with increased incidence of AF (Seiler et al., 2011; Mathew et al., 2014), LVH (Faul et al., 2011), HF (Ix et al., 2012), and mortality (Isakova et al., 2011) in a concentration-dependent manner in renal patients. Therefore, the prognostic value of high FGF-23 serum levels for predicting all-cause and cardiovascular mortality has been proposed in several clinical studies and meta-analyses in renal patients and especially ESRD dialysis (Gutiérrez et al., 2008; Isakova et al., 2011; Ix et al., 2012; Ärnlöv et al., 2013).

Faul et al. (2011) first noted that the pro-hypertrophic effect of FGF-23 is FGFR-dependent and mediated by the phospholipase C (PLC)γ-calcineurin-nuclear factor of activated T cells (NFAT). At the experimental level, pro-hypertrophic FGF-23 actions were found to occur in cardiomyocytes in an FGFR-dependent manner (Böckmann et al., 2019) but Klotho-independently (Faul et al., 2011). Andrukhova et al. (2014) proposed that FGF-23 increases sodium renal uptake Klotho-dependently, producing a volume overload and hypertension and finally leading to the development of LVH. By contrast, other authors have postulated that FGF-23 is not able to induce cardiac hypertrophy in the absence of hyperphosphatemia conditions (Liu et al., 2018). Moreover, it is not yet clear whether FGF-23 is the cause or a consequence of LVH. Thus, it has been demonstrated that transgenic mice that constitutively express active calcineurin A, the key pathway involved in pathological LVH, showed a significant increment in serum FGF-23 levels without any alteration of renal function (Matsui et al., 2018). Cardiomyocyte expression of FGF-23 under stress conditions has also been demonstrated by other authors. Specifically, it has been probed in different rodent models that the expression of FGF-23 is increased not only in bones but also in the heart as consequence of a myocardial infraction induction (Andrukhova et al., 2015). Furthermore, the expression of FGF-23 in cardiomyocytes has been observed to be increased by oncostatin M, a major mediator of cardiac remodeling (Richter et al., 2015).

Several studies have additionally shown a direct effect of FGF-23 on cardiac function. Different authors have demonstrated a relationship between high FGF-23 levels and predisposition to arrhythmia. First, it was found that high levels of FGF-23 were related to AF (Mathew et al., 2014; Lind et al., 2017). A prospective study found a strong association between FGF-23 and incident and prevalent AF in renal patients with mild-to-severe CKD (Mehta et al., 2016). This relationship was corroborated in experimental studies where the atrial immortal cell line HL-1 was incubated, with a high concentration (25 ng/mL) of FGF-23, inducing intracellular Ca^2+^ mishandling related to a pro-arrhythmogenic behavior (Kao et al., 2014). At the ventricular level, recent studies have demonstrated that FGF-23 is able to rapidly induce *in vivo* arrhythmic events by increasing the prevalence of premature ventricular contractions in healthy mice (Navarro-García et al., 2019). At the cellular level, an acute exposure to elevated FGF-23 concentration (100 ng/mL) has also been demonstrated to induce a pro-arrhythmogenic phenotype in isolated ventricular adult cardiomyocytes (Navarro-García et al., 2019). The increased incidence of pro-arrhythmogenic events in cardiac cells might be a consequence of the Ca^2+^ mishandling observed in cardiomyocytes exposed to high concentrations of FGF-23. In this work, FGF-23 effect on cardiomyocyte function is also shown to be FGFR-dependent. However, unlike the pro-hypertrophic effect, FGF-23-induced cardiomyocyte dysfunction is calmodulin kinase type II-dependent and independent of PLCγ (Navarro-García et al., 2019). In this sense, it is important to note that FGF-23 has been related to LV dysfunction even in the absence of LVH (Seiler et al., 2011), suggesting that FGF-23 might be activating different intracellular pathways on cardiomyocytes, likely depending on its concentration and exposure time. Furthermore, in the CKD mouse model of 5/6 nephrectomy which shows high serum FGF-23 but normal phosphate levels, cardiac dysfunction developed without any sign of cardiac hypertrophy (Navarro-García et al., 2020). Furthermore, recent works have proposed FGF-23, independently of phosphate levels, as one of the main factor responsible for the cardiac function alterations described in CKD patients (Isakova et al., 2018). All of this evidence proposes FGF-23 as a potential candidate to interconnect cardiac and renal systems, suggesting that it may be an important contributor to the cardiac burden experienced by renal patients.

### Klotho

Klotho is a protein mainly expressed in kidneys although it is also expressed in other organs such as the brain, pituitary gland, and ovaries (Kuro-o et al., 1997). There are two membrane Klotho isoforms, α-Klotho and β-Klotho. α-Klotho is the one that acts as the FGFR cofactor in the kidney (Urakawa et al., 2006). α-Klotho is mainly expressed in the distal tubules where phosphate reabsorption takes place (Kurosu et al., 2005). Thus, α-Klotho plays an important role in phosphate reabsorption in the kidney (Myrvang, 2012). α-Klotho binds to FGFR1 in the kidney through a receptor-binding arm that forms a complex with a groove in which FGF-23 fits with the N-terminal domain oriented toward FGFR1 and the C-terminal domain toward α-Klotho (Kuro-o, 2019). Membrane α-Klotho can be cleaved by secretases (ADAM10 and ADAM17) that release a soluble form of Klotho, named soluble Klotho or sKL (Chen et al., 2007). sKL functions as an endocrine factor for a widespread variety of surface glycoproteins, such as ionic channels, and growth factor receptors, such as insulin-like receptors involved in stress resistance (Wang and Sun, 2009) and aging control (Kuro-o, 2009), highlighting its implication in the aging process.

α-Klotho expression, and consequently sKL levels, are closely associated with kidney function. Several authors have shown that serum α-Klotho levels decrease as renal disease progresses (see Figure 1 and Table 2). Serum levels of sKL decrease from ∼670 pg/mL in healthy subjects to ∼300 pg/mL in ESRD patients. A small decrease of sKL values has also been found in AKI or CKD1 patients. As CKD progresses, sKL levels continue to decline quickly from the early stages of the disease onward until they reach values close to ∼280 pg/mL in CKD5 patients. Dialysis patients show values around ∼400 pg/mL, which is still significantly lower than the healthy population. The higher sKL levels in dialysis-treated patients might be due to these groups of patients comprising patients with different renal failure etiologies, meaning that some of them reach dialysis without passing through the five stages of CKD. On the other hand, it is well known that renal α-Klotho expression declines with aging. Indeed, the reduced expression of α-Klotho is implicated in age-related CKD development (Zeng et al., 2016). Furthermore, it has been demonstrated that higher levels of sKL are associated with a lower risk of declining kidney function (Drew et al., 2017). The role of α-Klotho in aging has been demonstrated in experimental animal models, as Klotho-deficient mice present reduced lifespan (Kuro-o et al., 1997) while mice overexpressing α-Klotho exhibited a longer mean lifespan (Kurosu et al., 2005). Among α-Klotho anti-aging effects, it is important to remark on the reduction of senescence in response to oxidative stress (Kim et al., 2019), an increment of cell survival in experimental uremia, and a protection against inflammation by decreasing expression and nuclear translocation of NFκB (Guo et al., 2018).

**TABLE 2:** Mean values of serum-soluble Klotho levels measured in pg/mL in healthy subjects and different renal patients.

Population	sKL levels (pg/mL)	References
Healthy	672.8 ± 78.7	Yamazaki et al., 2010; Yokoyama et al., 2012; Shardell et al., 2020; Yin et al., 2020; Zbroch et al., 2020; Salam et al., 2021
AKI	606.7 ± 55.3	Kim et al., 2016; Seibert et al., 2017
CKD1	547.8 ± 75.9	Kim et al., 2013; Khodeir et al., 2019; Bielesz et al., 2020; Yin et al., 2020
CKD2	517.1 ± 104.0	Kim et al., 2013; Seiler et al., 2014; Khodeir et al., 2019; Bielesz et al., 2020; Shardell et al., 2020; Yin et al., 2020
CKD3	508.9 ± 157.9	Kim et al., 2013; Seiler et al., 2014; Khodeir et al., 2019; Bielesz et al., 2020; Hughes-Austin et al., 2020; Yin et al., 2020
CKD4	343.5 ± 141.4	Kim et al., 2013; Seiler et al., 2014; Khodeir et al., 2019; Bielesz et al., 2020; Yin et al., 2020; Chen et al., 2021
CKD5	289.2 ± 160.6	Kim et al., 2013; Khodeir et al., 2019; Bielesz et al., 2020; Yin et al., 2020; Chen et al., 2021
Dialysis	435.0 ± 111.8	Yokoyama et al., 2012; Buiten et al., 2014; Fukasawa et al., 2014; Lima et al., 2014; Nowak et al., 2014; Otani-Takei et al., 2015; Desbiens et al., 2018; Valenzuela et al., 2019; Chan et al., 2020; Nakamura et al., 2020; Zbroch et al., 2020; Pizzarelli et al., 2021

*Mean values are obtained as the mean ± SD of the values found in the different cohorts studied. Those studies carried on diabetic or cardiovascular patient cohorts, with pediatric cohorts excluded.*

Some experimental studies have demonstrated that the absence of Klotho is associated with cardiovascular pathologies (Hu et al., 2011; Chen et al., 2016) and even with early unexpected death in mice (Takeshita et al., 2004). Klotho-deficient mice are characterized by hyperphosphatemia and enormously increased levels of serum FGF-23 (Kuro-o et al., 1997). Klotho-deficient mice also exhibit cardiac hypertrophy (Leifheit-Nestler et al., 2018) and cardiac dysfunction (Navarro-García et al., 2020). By contrast, Klotho-overexpressing mice are protected from cardiac dysfunction when CKD is induced (Navarro-García et al., 2020). However, it is unclear whether the cardiac alterations described in the absence of Klotho are a direct consequence of Klotho deficiency or induced by subsequent enormously increased serum FGF-23. At the clinical level, no association has been found yet between Klotho levels and cardiovascular mortality in the general population (Brandenburg et al., 2015), although it has been proposed Klotho as a predictor of all-cause mortality in the elderly population (Semba et al., 2011). In this line, a lower risk of cardiovascular disease development was found in elderly people with higher Klotho levels, even after adjusting for traditional cardiovascular risk factors (Semba et al., 2011). Moreover, lower sKL levels have been associated with increased mortality and cardiovascular events independently from other MBD-related factors in ESRD patients (Memmos et al., 2019). Several studies have also investigated the use of Klotho as therapeutic strategy owing its cardioprotective role. At the experimental level, cardiac dysfunction associated with reduced sKL serum levels has been prevented with enhanced sKL availability (Hui et al., 2017; Navarro-García et al., 2020). However, the mechanism through which sKL protects the heart remains unknown. It has been suggested that sKL protects the heart through the downregulation of the Transient Receptor Potential Cation Channel Subfamily C Member 6 (TRPC6) associated with HF (Xie et al., 2015; Hu et al., 2017; Han et al., 2020). Furthermore, it has been demonstrated that sKL prevents cardiac hypertrophy by direct regulation of several ion channels (Xie et al., 2012), or even in the context of uremic cardiomyopathy (Xie et al., 2015; Hu et al., 2017). Interestingly, it has been recently described that FGF-23-induced cardiac hypertrophy is also attenuated by sKL in mice. Han et al. (2020) proposed a possible switch on the signaling pathways induced by FGF-23 in the presence of sKL. The presence of Klotho might induce a change in the intracellular pathways activated in cardiomyocytes by FGF-23. Thus, in the absence of Klotho, FGF-23 activates the PLC-NFAT intracellular pathway, while when Klotho is available, the intracellular pathway activated by FGF-23 is the ERK pathway. Furthermore, enhanced Klotho availability has also been shown to protect against FGF-23-induced cardiac dysfunction *in vitro* (Navarro-García et al., 2019) and even in CKD mice (Navarro-García et al., 2020). Increased Klotho availability has additionally been found to protect against cardiovascular alterations developed in mice with reduced Klotho expression (Lim et al., 2012; Chen et al., 2016).

In conclusion, the cardioprotective role of Klotho, although promising, remains poorly understood. Some authors have proposed different mechanism for Klotho cardioprotection: (i) sKL would be able to bind to an unknown receptor activating an intracellular pathways that impede FGF-23 effects; (ii) in cells where the FGF-23 effect is Klotho-independent (as occurs in cardiomyocytes), the presence of sKL would block FGF-23 signaling once it binds to the FGFR, probably the FGFR4 isoform; or (iii) sKL might function as a soluble “*lure*” for FGF-23, binding to FGF-23 at the circulatory level and impeding its subsequent interaction with FGFR (Grabner and Faul, 2016; Navarro-García et al., 2019). More studies are needed to clarify Klotho’s actions and its relationship with FGF-23 in the heart from a functional perspective and especially in a uremic cardiomyopathy setting.

## Fibroblast Growth Factor-23-Klotho Axis as Therapeutic Target in Renal Disease

**FIGURE 2:**
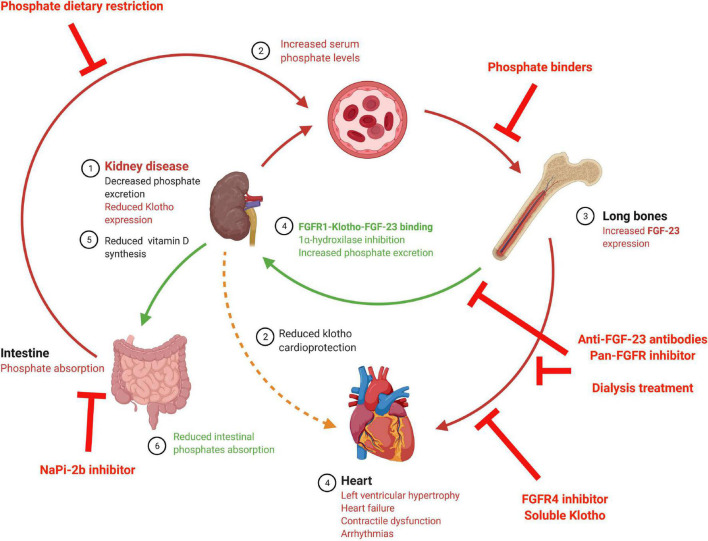
Schematic diagram of possible strategies to impede deleterious FGF-23 cardiac effects. The above illustration shows the FGF-23 synthesis stimuli and FGF-23 effects, along with the different treatments studied to avoid FGF-23 cardiac effects. FGFR, Fibroblast growth factor receptor; FGF-23, fibroblast growth factor 23; NaPi-2b, Sodium-Phosphate cotransporter 2b.

As reviewed recently by Verbueken and Moe (2021) there are different treatments designed to block high FGF-23 levels that vary depending on the target (see Figure 2 and Table 3), although not all of these are recommended for use with renal patients. The first target is to decrease FGF-23 production. FGF-23 production is lowered by decreasing serum phosphate levels by reducing dietary phosphate intake (Calvo et al., 2019), a crucial recommended management in renal patients. It has been shown that phosphate dietary restrictions reduce FGF-23 serum load in CKD patients (Sigrist et al., 2013; Chang et al., 2017), although no effect has been observed in the healthy population (Larsson et al., 2003). At experimental level, dietary phosphate restriction has been also demonstrated to reduced systemic FGF-23 levels in 5/6 nephrectomized rats with a significant improvement of kidney function (Duayer et al., 2021) and prevention of cardiac fibrosis and hypertrophy (Finch et al., 2013). However, Xie et al. (2015) has described no effect on serum FGF-23 levels and no prevention of cardiac hypertrophy after dietary phosphate restriction. Serum phosphate load can also be reduced clinically using phosphate binders, a typical therapy in renal patients. Several clinical and experimental studies have demonstrated that pathological synthesis of FGF-23 can be prevented with a phosphate binder in uremic conditions (Ketteler et al., 2019; Neven et al., 2020; Mason et al., 2021) although other studies has no probed any changes in serum FGF-23 levels after the use of these treatments (Chue et al., 2013; Ruggiero et al., 2019; Toussaint et al., 2020). When used in experimental animal models of CKD, phosphate binder reduced serum FGF-23 levels (Ghorbanihaghjo et al., 2018) with a significant reduction in aortic calcification (Finch et al., 2013; Wu-Wong et al., 2016). Moreover, serum phosphate levels can be controlled through the reduction of intestinal phosphate absorption by inhibiting the NaPi-2b cotransporter. In this sense, nicotinamide and niacin, both NaPi-2b inhibitors, have been demonstrated to reduce FGF-23 serum levels in a controlled trial of ESRD (Liu et al., 2020). However, this effect has not been found in clinical trial with stage 3 CKD patients treated with niacin (Rao et al., 2014) or nicotinamide (Ix et al., 2019). Furthermore, in an experimental mouse model of CKD, serum FGF-23 levels increased in long term after the use of this inhibitors (Thomas et al., 2019).

**TABLE 3:** Therapeutic strategies directed to decreased serum FGF-23 levels or to avoid FGF-23 effects.

Treatment	Study	Specie	Renal disease	Serum FGF-23	Outcomes
**Decrease FGF-23 synthesis**					
Reduced Pi intake	Chang et al., 2017	Human	Early CKD	Unchanged	No changes in albuminuria
	Sigrist et al., 2013	Human	CKD	Reduced	
	Xie et al., 2015	Mouse	CKD (5/6 Nfx)	Unchanged	No prevention of cardiac hypertrophy
	Finch et al., 2013	Rat	CKD (5/6 Nfx)	Reduced	Reduced aortic calcification Partially reduced cardiac fibrosis Prevented cardiomyocyte hypertrophy
	Duayer et al., 2021	Rat	CKD (5/6 Nfx)	Reduced	Improved kidney function
Pi binders	Ruggiero et al., 2019	Human	CKD	Unchanged	Unchanged Klotho levels Ameliorated inflammation Improved dyslipidemia
	Mason et al., 2021	Human	CKD (stage 3-4)	Reduced	Decreased inflammatory levels Improved vascular calcification
	Ketteler et al., 2019	Human	ESRD	Reduced	Improved bone metabolism
	Chue et al., 2013	Human	CKD (stage 3)	Unchanged	No changes in left ventricular mass, systolic and diastolic functions, or arterial stiffness
	Toussaint et al., 2020	Human	CKD (stage 3b-4)	Unchanged	No amelioration of arterial stiffness Non-improved of aortic calcification
	Ghorbanihaghjo et al., 2018	Rat	CKD (adenine diet)	Reduced	
	Finch et al., 2013	Rat	CKD (5/6 Nfx)	Reduced	Non-reduced aortic calcification Reduced mortality Non-reduced cardiac fibrosis Increased cardiomyocyte area
	Neven et al., 2020	Rat	CKD (adenine diet)	Reduced	Improved kidney function Decreased bone fibrosis
	Wu-Wong et al., 2016	Rat	CKD (5/6 Nfx)	Reduced	Reduced aorta calcification
NPT2 inhibitiion	Rao et al., 2014	Human	CKD	Unchanged	
	Ix et al., 2019	Human	CKD (stage 3b-4)	Unchanged	
	Thomas et al., 2019	Mouse	CKD (5/6 Nfx)	Increased in the long term	Reduced PTH
**Increased FGF-23 clearance**					
Dialysis	Kawabata et al., 2020	Human	ESRD	Reduced	Unchanged Klotho levels
	Tiong et al., 2021	Human	ESRD	Reduced	
**FGF-23 neutralization**					
Anti-FGF-23 antibody	Sun et al., 2015	Mouse	CKD (5/6 Nfx)	Reduced	Improved bone quality
	Shalhoub et al., 2012	Rat	CKD (High Pi diet)	Unchanged	Increased aortic calcification Increased mortality
**Blockage of FGFR**					
Pan-FGFR antibody	Di Marco et al., 2014	Rat	CKD (5/6 Nfx)	Unchanged	Improved cardiac structure and function
	Faul et al., 2011	Rat	CKD (5/6 Nfx)	Unchanged	Attenuation of cardiac hypertrophy
Anti-FGFR4 antibody	Grabner et al., 2017	Rat	CKD (5/6 Nfx)	Unchanged	Reduced cardiac hypertrophy
**Klotho treatment**					
Recombinant Klotho	Navarro-García et al., 2020	Mouse	CKD (5/6 Nfx)	Unchanged	Prevention of cardiac dysfunction
	Suassuna et al., 2020	Rat	CKD (5/6 Nfx)	Unchanged	Prevented cardiac hypertrophy and fibrosis
Transgenic Klotho expression	Xie et al., 2015	Mouse	CKD	Unchanged	Reduced cardiac hypertrophy and fibrosis

*Works that evaluate serum FGF-23 levels in renal disease in clinical or experimental studies with any of the treatments reviewed in this study have been included in this table.*

*Pi, phosphate; CKD, chronic kidney disease; ESRD, end-stage renal disease; FGF-23, fibroblast growth factor-23; FGFR, fibroblast growth factor receptor; Nfx, nephrectomy; NPT2, sodium-phosphate cotransporter 2.*

Increased FGF-23 serum levels can be also corrected by increasing FGF-23 clearance. In this sense, it has been described that dialysis reduced FGF-23 levels (Kawabata et al., 2020; Tiong et al., 2021), possibly as a consequence of reduction in phosphate serum levels (Chan et al., 2020). In this case, FGF-23 reduction might be a direct effect of dialysis, as different FGF-23 reductions have been found depending on the type of hemodialysis in use (Patrier et al., 2013). It is important to note that FGF-23 levels in dialysis-dependent renal patients are extremely high despite the dialysis process, and that these patients are usually treated with phosphate binders and under dietary phosphate restrictions. Consequently, recent approaches are exploring other mechanisms to block the deleterious effects of FGF-23 in renal patients. To this aim, some researchers have studied experimentally the effect of FGF-23-neutralizing antibodies. In spite of reducing serum FGF-23 levels improving bone quality in CKD mice (Sun et al., 2015), anti-FGF-23 antibodies treatment was found to significantly augment phosphate levels, vascular calcification, and death risk in an experimental CKD model (Shalhoub et al., 2012). A complete FGF-23 function inhibition would also block its phosphaturic action, which is still important when renal function is not completely lost. Thus, completely blocking the effects of FGF-23 would increase phosphate levels in early stage renal patients, likely with undesirable consequences. It is probably for this reason that no studies of anti-FGF-23 antibodies have carried out in renal patients thus far to date.

Another proposed strategy is the blockage of deleterious off-target FGF-23 action (such as that in the heart) by specific FGFR inhibitors. In this line, a pan-FGFR inhibitor has been shown to impede cardiac hypertrophy (Faul et al., 2011; Di Marco et al., 2014) and dysfunction (Navarro-García et al., 2019) induced by FGF-23. However, the inhibition of all FGFR would also block FGF-23 phosphaturic actions in the kidney, mediated by FGFR1, which may in turn increase serum phosphate levels promoting the synthesis of FGF-23. Since FGFR4 has been proposed as the necessary mediator of cardiac FGF-23 effects (Grabner et al., 2015, 2017) a better strategy to prevent the deleterious cardiac effects of FGF-23 would be the use of specific FGFR4 blockers. The use of a specific FGFR4 inhibitor would avoid the inhibition of the physiologic phosphaturic effect of FGF-23 on the kidney mediated by FGFR1. However, no FGFR inhibitors have been used in humans with renal disease so far.

**FIGURE 3:**
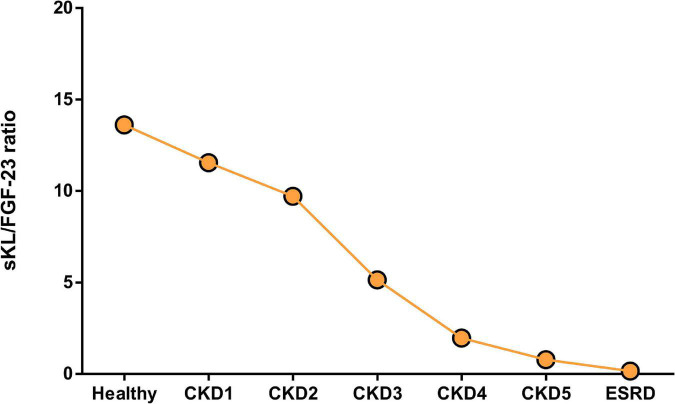
Ratio between serum sKL and FGF-23 levels in healthy population and renal patients alongside CKD and dialysis. Ratio was obtained between sKL and FGF-23 from the same cohort of patients included in Tables 1, 2; only studies where both values of these markers were reported were used for this graph.

As discussed above there are different approaches to reduce FGF-23 actions preventing deleterious actions on the heart. However, all of them could block the actions of FGF-23, not only the pathological but also de physiological phosphaturic action. The loss of the FGF-23 phosphaturic action would increase even more the serum phosphate levels in those renal patients with remaining kidney function that would be an important side effect to be considered in those treatments. New therapies to prevent cardiac effect of FGF-23 without impeding FGF-23 phosphaturic effect are needed. Thus, the use of sKL as a therapeutic strategy to block the deleterious effects of FGF-23 on the heart without altering its physiological phosphaturic function is becoming increasingly important nowadays. In this sense, Law et al. (2020) has reviewed recently different approaches to increase Klotho levels as a possible therapeutic strategy to prevent cardiac alterations described under uremic conditions. Several experimental studies have demonstrated that sKL treatment prevents heart alterations found following renal dysfunction. Thus, sKL treatment was found to prevent FGF-23-induced cardiac hypertrophy (Han et al., 2020; Suassuna et al., 2020) and dysfunction (Navarro-García et al., 2020) in experimental CKD models. In this line, Klotho overexpression has been shown to prevent cardiac alterations in a uremic milieu (Navarro-García et al., 2020). Nevertheless, some authors propose that Klotho’s cardioprotective role is FGF-23- and phosphate-independent (Xie et al., 2015). Thus, maintaining adequate Klotho levels might represent a new therapeutic strategy to avoid the cardiac dysfunction described in renal patients, including those cardiac effects mediated directly by FGF-23, while at the same time guaranteeing its physiological phosphaturic action. Studies on the inhibition of FGF-23 effects on the heart in the presence of sKL remark on the importance of the role of not only FGF-23 but also sKL in the heart. In the present review, we propose the balance between FGF-23 and sKL availability as a new prognostic tool for renal patients (Figure 3). Decreased Klotho availability together with increased systemic FGF-23 levels should be taken into consideration as a warning sign for increased risk of deleterious cardiac prognosis in renal patients. Whether a ratio sKL/FGF-23 is used as a tool to characterize the balance between both mineral components in renal patients, we can observe a significant reduction of this ratio from the early stages of CKD. In early CKD, the reduction of sKL/FGF-23 ratio occurs mainly due to the reduction of sKL values, as FGF-23 values are higher than in the healthy population but similar from CKD1 to CKD3. However, from CKD4 onward, the reduced sKL/FGF-23 must be the consequence of the highly increased serum FGF-23 levels in these patients. The sKL/FGF-23 ratio could be used as a tool to choose an adequate treatment that impedes FGF-23 side effects on organs other than the kidney, such as the heart. Thus, in those CKD stages where the misbalance is the result of decreased sKL levels, it would be useful to increase sKL values; exogenous Klotho administration could be an adequate option under these circumstances. However, in advanced CKD stages where the FGF-23 levels are enormously high, treatment directed at reducing FGF-23 synthesis could be more adequate for renal patients.

## Concluding Remarks and Future Perspectives

In conclusion, there is growing evidence to support the important role played by the FGF-23-Klotho axis in the CRS. Thus, FGF-23 and Klotho should be considered not only as important biomarkers in renal patients to evaluate cardiovascular events and mortality risks, but also as a therapeutic target. Many human and experimental studies have demonstrated that high levels of FGF-23 or low levels of sKL are linked with cardiovascular risk and mortality, although few of these have considered the balance between them as a possible therapeutic target. Recent studies have shown that increased Klotho availability might protect the heart even in high FGF-23 conditions with and without hyperphosphatemia and with no renal modifications, indicating that Klotho may be the best therapeutic target to prevent FGF-23’s deleterious effects on the heart identified thus far. However, more studies are needed to analyze more adequate strategies to block FGF-23 action and enhance Klotho availability, especially in humans.

## Author Contributions

JAN-G and GR-H designed, conceptualized the review, and wrote the first draft of the manuscript. LG-L, MF-V, and LR participated in different sections of the review. All authors revised and approved the final version of the review.

## Conflict of Interest

The authors declare that the research was conducted in the absence of any commercial or financial relationships that could be construed as a potential conflict of interest.

## Publisher’s Note

All claims expressed in this article are solely those of the authors and do not necessarily represent those of their affiliated organizations, or those of the publisher, the editors and the reviewers. Any product that may be evaluated in this article, or claim that may be made by its manufacturer, is not guaranteed or endorsed by the publisher.
